# Crystallization properties of arsenic doped GST alloys

**DOI:** 10.1038/s41598-019-49168-z

**Published:** 2019-09-10

**Authors:** Vinod E. Madhavan, Marcelo Carignano, Ali Kachmar, K. S. Sangunni

**Affiliations:** 1 Qatar Environment and Energy Research Institute, Hamad Bin Khalifa University, Qatar Foundation, P. O. Box 34110, Doha, Qatar; 2 Department of Physics, Indian Institute of Science, Bangalore, 560012 India

**Keywords:** Information storage, Electronic properties and materials

## Abstract

We present the enhanced properties observed in the phase change memory alloy Ge_2_Sb_2_Te_5_ (GST) when doped with arsenic. Although arsenic is known as a toxic element, our observations show that significant improvement can be obtained in GST systems on thermal stability, transition temperature between amorphous and crystalline phases and switching behaviors when doping with arsenic. Though both the GST and arsenic doped GST are amorphous in the as-deposited state, only GST alloy turns to crystalline NaCl-type structure after annealing at 150 °C for 1 h. Results from the resistance versus temperature study show a systematic increase in the transition temperature and resistivity in the amorphous and crystalline states when the arsenic percentage in the GST alloy increases. The crystallization temperature (T_c_) of (GST)_0.85_As_0.15_ is higher than the T_c_ observed in GST. Optical band gap (E_opt_) values of the as-deposited films show a clear increasing trend; 0.6 eV for GST to 0.76 eV for (GST)_0.85_As_0.15_. The decreases in E_opt_ for the samples annealed at higher temperatures shows significant optical contrast between the as-deposited and annealed samples. Though all (GST)_1−x_As_x_ alloys show memory switching behaviors, threshold switching voltages (V_T_) of the studied alloys show an increasing trend with arsenic doping. For (GST)_0.85_As_0.15_, V_T_ is about 5.2 V, which is higher than GST (4.0 V). Higher transition temperature and higher threshold switching values show arsenic doping in GST can enhance the memory device properties by improving the thermal stability and data readability. Understanding the doping effect on the GST is important to understand its crystallization properties. Structure properties of amorphous GST, Ge_2_Sb_2−0.3_As_0.3_Te_5_ and (GST)_0.85_As_0.15_ models were studied using first principles molecular dynamics simulations, compared their partial radial distribution functions, and q parameter order. Arsenic doping into GST features interesting structural and electronic effects revealed by the radial distribution functions, q order parameter and band gap value, in line with the experimental findings.

## Introduction

Among chalcogenide compounds, Ge-Sb-Te (GST) alloys are phase-change materials that possess superior properties for memory applications and are currently used in optical and electrical rewritable data storage devices. The phase change memory (PCM) properties were first observed by Ovshinski in evaporated Te-As-Si-Ge films^1^. Nowadays GST materials are used in Phase change Random Access Memory (PRAM) devices for non-volatile data storage applications owing to their compatibility with complementary metal oxide semiconductor (CMOS) technology^2,3^. GST alloys are characterized by the strong contrast between high resistance amorphous states and low resistance crystalline states that can be switched back and forth using a nanosecond laser/current pulse. The suitable properties which make these materials attractive for PRAM applications are fast reversible phase-change, high data storage density, long endurance, long cycles of operation and stability in an ample range of ambient conditions^4–6^. On-chip photonic memory elements employing phase-change materials have been investigated by exploiting their high contrast optical properties in photonic integrated circuits^7^.

Though GST is a well-studied system, there are still a lot of hurdles and uncertainties in the structural aspects of the material and its fabrication and stability. Many ongoing studies aim to improve the material properties by doping with suitable elements and to overcome practical application oriented problems like high reset current, crystallization speed, thermal stability in the amorphous state, etc^8–11^. The addition of suitable impurities may bring enhancements to the GST materials that could be useful for PCM applications. Various doping elements such as Oxygen, selenium, nickel, carbon, indium, silicon, titanium are introduced to GST^12–17^. It has been found that the Si doping (4.1 at.%) reduces the writing current in GST, through there were Si phase separation^16^. Titanium has been doped in GST up to 1.23%, which increases the crystallization temperature and increased the thermal stability of the FCC phase. Also, it restrained the phase separation between FCC and HCP phases as the Ti concentration increases^17^. Oxygen reacted with Ge and Sb and form oxides keeping a Te depleted in the GST surface. By doping with selenium, resistance, threshold voltage for switching and thermal stability of the GST alloy is increased. Low percentage (2%) of nickel brings better electrical properties without affecting the lattice structure of undoped GST. When carbon dopants were introduced, high Tc, low RESET current and low resistance drift were observed. Indium doping (upon 3 wt. %), changed Tc, resistivity, optical band gap width etc. in GST thin films. To the best of our knowledge, there is no published work exploring the effect of arsenic as an additive in GST and therefore it is important to study the changes in the structural and phase-change properties caused by this additive. We have prepared a series of films of (GST)_1−x_As_x_ with different As content. It is interesting to investigate what structural changes are affected in the GST system upon the addition of As. One possibility is that As replaces Ge because of their comparable atomic radius and atomic weight (115 pm and 74 amu, respectively for As; and 125 pm and 72.64 amu, for Ge). However, the outer electronic configuration of Ge is different from that of As, so an alternative chance is As replacing Sb owing to their similar chemical character. Since the electro negativity of As (2.18 in the Pauling scale) is higher than that of Ge (2.01), it may bond with Ge to replace Ge-Te bonds. It is known that the GeTe (50:50) exact ratio requirement is relaxed by doping with Sb to form another superior alloy such as Ge_2_Sb_2_Te_5_. This allows for the change in the Te content between 45 to 55 at.% with enhancement in properties^18^. On the other hand, the addition of As can cause several changes in the GST system since it belongs to the same group of Sb. As has lower density (5.72 g/cc for As, 6.62 g/cc for Sb), smaller atomic radius (133 pm for Sb) and atomic weight (121.75 amu for Sb) than Sb. Most importantly, the electro negativity of As is higher than that of Sb (2.05 Pauling scale). It is also well known that As is good glass former. All these factors will contribute to determine the structural network and therefore the properties of the (GST)_1−x_As_x_ films. The aim of this work is to see how the As affects the crystallization ability and the resistance contrast of GST phase-change memory materials. In addition to the experimental work, we perform a first principles molecular dynamics study based on previously published models for undoped GST and (GST)_1−x_As_x_. The combination of experiments and simulation work result in a comprehensive picture of the As effects on GST.

## Experimental Details

Thin films of (GST)_1−x_As_x_, with x = 0, 0.02, 0.10 and 0.15 in at. %, were prepared from a stoichiometric target using thermal evaporation onto glass substrates. During the deposition process (at normal incidence), the substrates were suitably rotated in order to obtain films of uniform thickness. The thicknesses of the films were 500 nm. Thickness was measured with a stylus profiler (Dektak) as well as cross-sectional scanning electron microscopy (Quanta). The composition of the deposited films was analyzed by energy dispersive X-ray spectrometry (EDS) with a variation of ± 5 at% for Sb + Te and ± 13 at% Ge + As with respect to the bulk targets. The thin films were annealed at selected temperatures in a vacuum of 10^−5^ mbar for 1 h using a resistive heater. The amorphous state of the as-deposited (AD) and the crystalline structure of the annealed films were checked by X-ray diffractometry (Bruker D8 Advance, Cu_Kα_, λ = 1.54 Å). Transmission spectra of the films in NIR range (500–1850 nm) were obtained using a PerkinElmer Lambda 750 UV-VIS-NIR spectrometer. The sandwich type devices were fabricated on Pt coated glass substrates by depositing (GST)_1−x_As_x_ films followed by Al contact top electrode by thermal evaporation. The diameter of the active area of the device is 350 micron and the geometry was Pt(100 nm)/PCM(500 nm)/Al(300 nm). We used a probe station with Agilent Device Analyzer B1500A in order to perform the switching studies. The current swept between the upper Al and lower Pt electrodes and the corresponding voltages were measured. The Agilent Device Analyzer was also used to perform four-point probe resistance measurements by the van der Pauw method in order to study the effect annealing temperature on the films. Each resistance value is an average of three measurements.

## Computational Details

We have performed molecular dynamics (MD) simulations using the hybrid Gaussian and plane waves method (GPW) as implemented in the Quickstep module of the CP2K package (V 5.1)^19–21^. For all cases the simulations were run under NVT conditions, with the temperature controlled by a Nosé-Hoover thermostats with 3 chains and a time constant of 50 fs^22–24^. The electronic structure properties were calculated using the PBE^25^ functional with the empirical correction from Grimme scheme (DFT-D3)^26,27^ to account for the dispersion interactions, which were shown to be important for liquid Ge_15_Te_85_^28^, glassy GeTe_4_^29^ and amorphous Ge_2_Sb_2_Te_5_^30^. Kohn-Sham orbitals are expanded in a double-zeta polarization Gaussian-type basis set for the all atoms (MOLOPT-DZVP-SR-GTH for Ge, Sb, As, Te), and we employed the norm-conserving GTH pseudopotentials^31^. The auxiliary plane wave (PW) basis set was defined by the energy cutoff of 300 Ry, and with a relative cutoff of 50 Ry. All the simulations were carried out under Periodic Boundary Conditions (PBC) on the considered models. The time step of the integration of the dynamic equations was set to 2 fs on all the quenching/annealing steps. The Brillouin zone was sampled at Γ point of the supercell. The calculation of the band gap was performed with varying percentage of the short range Hartree-Fock exchange, combined with the PBE functional as explained in ref.^32,33^.

We focused on three model systems containing 459 atoms: (i) The first model corresponds to the reference Ge_2_Sb_2_Te_5_ (102 Ge, 102 Sb and 255 Te atoms) material, referred to simply as GST. (ii) The second model is for a doped alloy obtained by direct *substitution* of Sb by As atoms (102 Ge, 15 As, 87 Sb and 255 Te atoms) that corresponds to 3% of As and is referred to by the formula Ge_2_Sb_2−0.3_As_0.3_Te_5_. (iii) The third model is for a doped alloy obtained by *proportional replacement* of the Ge, Sb and Te atoms by As according to its pure GST relative composition resulting in (GST)_1−x_As_x_ with x = 15% (86 Ge, 72 As, 86 Sb, 215 Te). The GST model was created following the work of S. Caravati *et al*.^34^. The three model systems were simulated in a cubic cell with an edge length of 24.4 Å.

We studied the formation of the amorphous system from the melt following the procedure of Ronneberger *et al*.^35^. The method starts with a 5 ps equilibration run for the melt at 3000 K, which is well above the melting point (~1000 K). Next, we perform a cooling to 1000 K at a rate of 100 K ps^−1^. We proceed with an extra equilibration run at 1000 K for 3 ps. The amorphous state is obtained by quenching down the three models to 300 K, at a quenching rate of 15 K ps^−1^. After that, we equilibrated the cooled structure for another 2 ps at 300 K, and we further extended the simulations for another 40 ps in order to obtain a production trajectory. For the ground state calculations, the three models were quenched to their final fully optimized structures.

## Results and Discussion

### XRD studies

**Figure 1:**
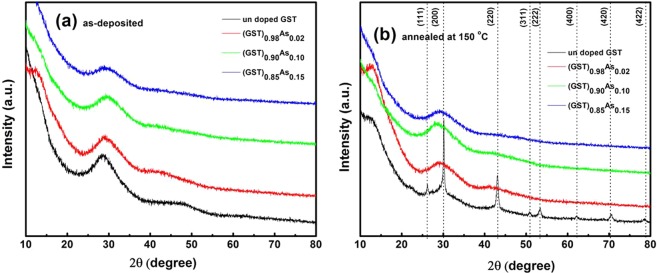
(**a**) XRD of the (Ge_2_Sb_2_Te_5_)_1−x_As_x_ (x = 0, 0.02, 0.10, 0.15) as-deposited films and (**b**) annealed at 150 °C.

Figure 1(a) shows the X-ray diffraction patterns for the As doped GST thin films. The absence of sharp diffraction lines in the X-ray patterns indicates that the as-deposited films are amorphous in nature. Figure 1(b) shows that at 150 °C only the pure GST film has crystallized. The crystalline structure is identified as rock salt with space group Fm-3m [04-011-9024@ 2012 ICDD] and the peaks are indexed against hkl planes. Crystallization of the (GST)_1−x_As_x_ films were observed at annealing temperatures higher than 150 °C as it is shown by the XRD patterns displayed on Fig. S1.

Since the structural modification is by the addition of As to GST, As may remain in the interstitials or vacancies or can replace existing elements in the GST alloy and can affect phase change properties. In the crystalline state of GST, As most probably replaces or substitutes Sb due to the same valence electronic configuration. Another possibility of the As addition is the phase separation of the As itself. However, such segregations were not observed in the crystalline state, there were no impurity peaks due to a separate As phase detected in the XRD study. Similar doping has been reported in GST system such as (Ge_2_Sb_2_Te_5_)_1−x_Ag_x_ where the Ag atoms remain in the interstitials of the GST alloy without affecting the basic structure^11^.

### UV-VIS transmission studies

**Figure 2:**
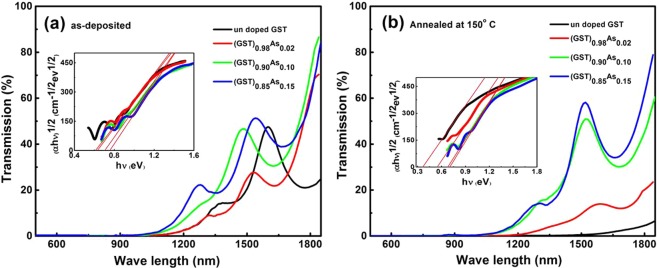
Transmission spectra of (GST)_1−x_As_x_ thin films (**a**) as-deposited and (**b**) annealed at 150 °C. Inset show the corresponding band gap spectra.

Transmission spectra of the as-deposited (GST)_1−x_As_x_ films are shown in Fig. 2(a). With increasing As content in the GST alloy, the absorption edge is shifted to a lower wavelength. The transmission minimum is at 1100 nm for GST and 1000 nm for 15% As doped GST film indicating the changes in the band gap. The transmission is reduced for GST alloy due to the crystallization and the transmission edge is red-shifted further to 1600 nm as displayed in Fig. 2(b). However, with 10% and 15% As content the GST alloys are still not crystallized as becomes evident from the high transmission percentage. The optical band gap of each alloy is calculated from the transmission spectra using a Tauc plot where the absorption coefficient α > 10^4^ cm^−1^^36^. The transition is indirect and the obtained band gap values are given in Table 1. The samples annealed at higher temperatures (200 and 300 °C), show that the transmission is further reduced indicating the structural modifications of GST due to the As addition. The band structural modifications due to arsenic addition is evident in the shift of transmission edge in (GST)_1−x_As_x_ films which are complementary to XRD studies.

**Table 1:** The optical band gap and threshold switching voltages of (GST)_1−x_. As_x_ films, as determined from the Tauc plots and I-V measurements.

(GST)_1−x_As_x_	x = 0.0	x = 0.02	x = 0.10	x = 0.15
E_opt_(eV)_**as-deposited**_	0.61	0.63	0.71	0.76
	Amorphous state			
E_opt_(eV)_**150 °C**__**annealed**_	0.37	0.55	0.68	0.70
	Crystalline state	Amorphous state		
Threshold Voltage (V_T_)	4.0	4.4	4.7	5.2

It is found that the E_opt_ increases from 0.61 eV for GST to 0.76 eV for (GST)_0.85_As_0.15_ in the as-deposited films. This means that the As addition rearranges the band structure in such a way that the band gap widens. After annealing the films at 150 °C, a considerable change in the band gap for the GST (ΔE_opt_ = 0.24 eV) is observed as the result of the crystallization. For the As doped films, the annealing process results in a smaller band gap, however the changes are less significant as in the case of pure GST film. The doped films are still not crystallized completely at 150 °C as evident in the XRD patterns and transmission spectra. The addition of As creates localized defect states in the band gap since it is acting as an impurity in the system. Also the co-ordination number of this system defines the networks as rigid or over coordinated^37^ and therefore the impurity levels cause the changes in the activation energy and band gap. This is one of the reasons for increased crystallization resistances.

GST/metal planar structure interfaces, if realized as an absorber layer for solar cells, could bring a significantly high absorption in the infrared region. Furthermore, to achieve higher absorption in the visible region for photovoltaic (PV) applications, arrays of metallic resonators and GST can be fabricated to exploit the combined effect of Plasmon resonance and the highly dispersive characteristics of the crystalline GST layer^38^. Arsenic doping to GST could help to tailor the band gap properties in order to achieve an overall higher absorption in the near infrared region of the solar spectrum for PV applications especially in tandem devices.

### Resistance Vs temperature studies

**Figure 3:**
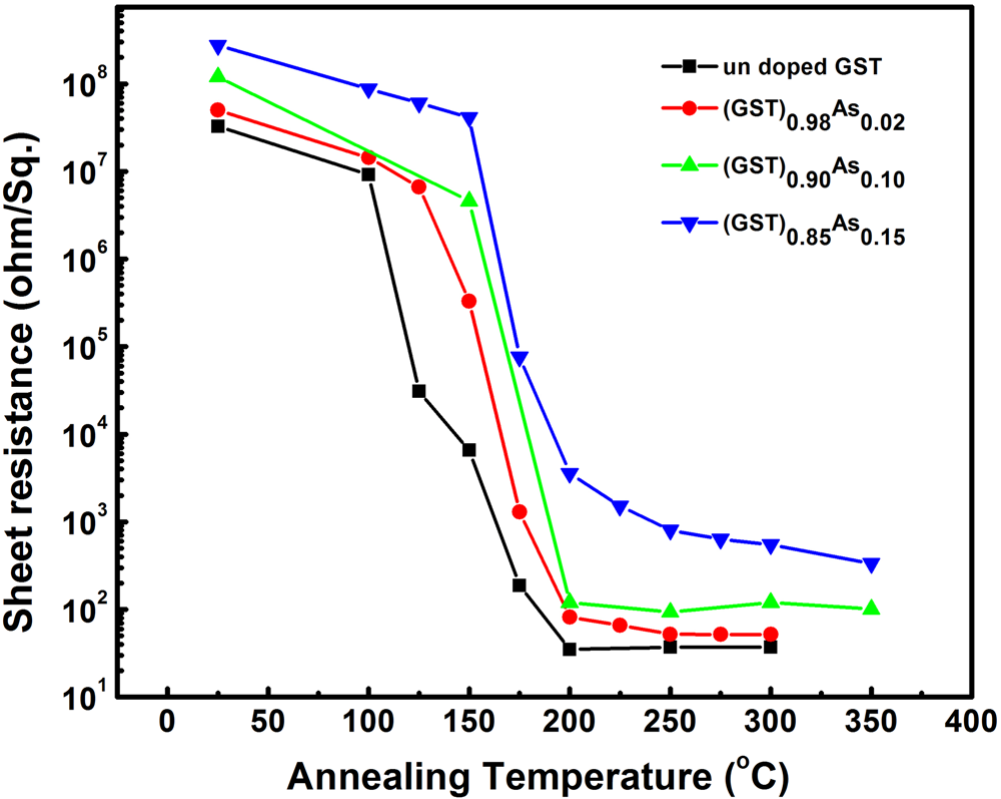
Resistance as a function of temperature for the pure GST and the selected (GST)_1−x_As_x_ films. The measurements are performed after the films have undergone isothermal annealing at selected temperatures in vacuum.

The isothermal resistance of different (GST)_1−x_As_x_ samples measured after annealing at different temperatures for 1 h to understand the phase transition behavior are given in Fig. 3. It can be seen that the resistance decreases with increasing annealing temperature above 100 °C. A steep decrease in resistance indicates the onset of phase changes in the films. In the pure GST film, the resistances show a clear decrease at 125 °C. For the 2% As added GST films, the phase change signal is visible at 150 °C. In 10% and 15% As doped GST, the change occurs at even higher temperatures. The film resistance shows that the transition temperature of the alloys increases with As addition, in line with what we concluded from the XRD and transmission studies. A higher transition temperature increases the amorphous stability and archival life time of the memory state^39^. The increase in the transition temperature is then associated with the As content of the alloy. Also, it is evident from Fig. 3 that the resistance of the as-deposited (amorphous) and crystallized films increases with higher As content. The resistance contrast also increases with higher As content. Generally, it is expected a resistance drop upon crystallization due to a reduction in electron scattering at grain boundaries. In this case the As dopant in GST may act as scattering centers at the grain boundaries and associated phase formation causes this higher value of resistances in the crystallized films. A larger resistance of the crystalline state is advantageous as it can reduce the writing current in the memory device. However, it is important to note here that the resistance contrast of about 5 orders of magnitude in the case of GST is decreased to four orders of magnitude in the case of 15% arsenic added GST film.

### Device switching studies

**Figure 4:**
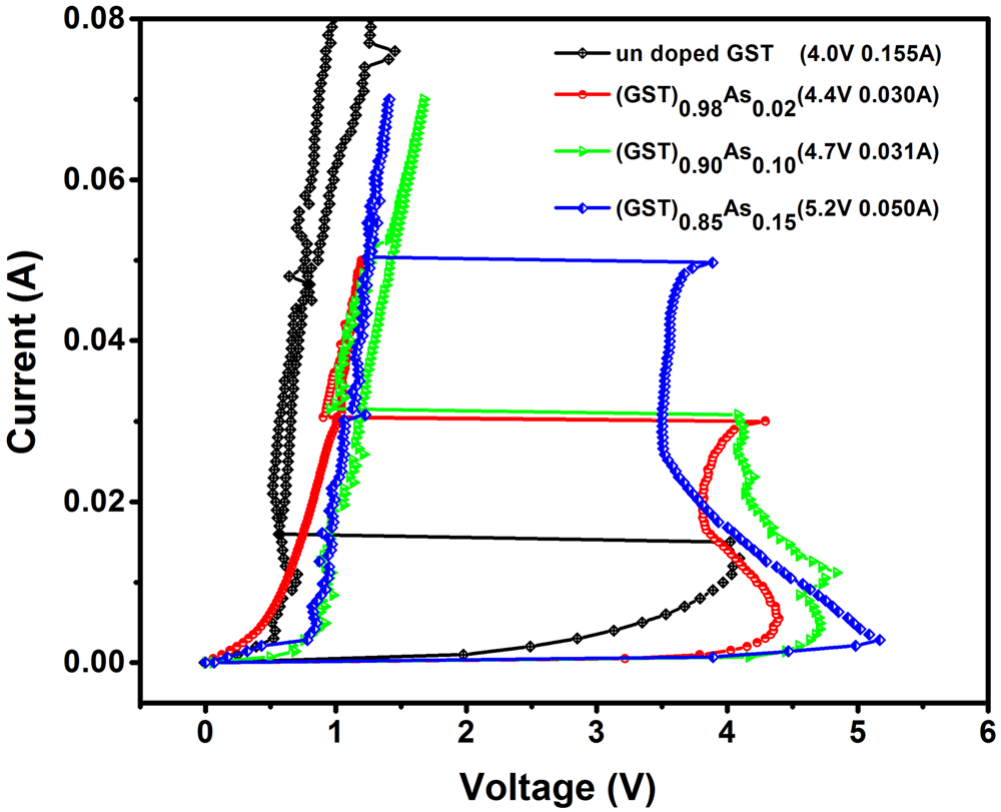
Switching studies of (GST)_1−x_As_x_ based memory devices. The device structure is a planar, circular sandwich geometry with Pt(100 nm)/PCM(500 nm)/Al(300 nm). The diameter of the active area of the device is 350 μm.

The current-voltage switching behaviors of the (GST)_1−x_As_x_ based memory devices are shown in Fig. 4. All devices display a memory switching behavior with different threshold voltages (V_T_). The V_T_ of each device is indicated in the legends of Fig. 4. Generally, the as-deposited films are in the amorphous (OFF) state and show a very high resistance^40^. Above a voltage value (threshold voltage V_T_), the material switches to a highly conducting crystalline state (ON) and the current increases. At this point of time, atoms rearrange by themselves due to the heating and the material crystallizes. As shown in Fig. 4, the V_T_ for GST device is 4.0 V and for (GST)_0.85_As_0.15_ device, it is 5.2 V. A systematic increase in the V_T_ is observed with increasing As content in the alloy. The increased crystallization temperature due to the As addition is the reason for increased V_T_ and indirectly this reflects the increased thermal stability. A higher V_T_ represents the higher activation energy required for the system to switch to the crystalline ON state.

### Structural properties of the amorphous state for GST, Ge_2_Sb_2−0.3_As_0.3_Te_5_ and (GST)_0.85_As_0.15_

**Figure 5:**
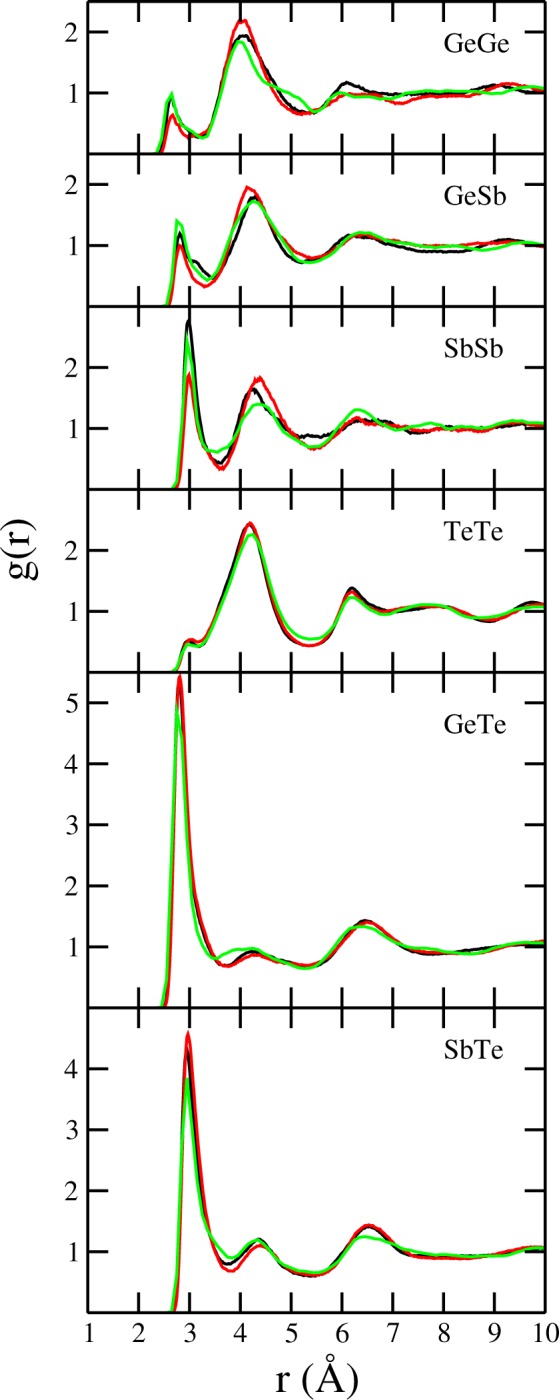
Pair radial distribution functions, g(r), for the three amorphous models. The black lines correspond to the pure GST (GST), the red lines are for Ge_2_Sb_2−0.3_As_0.3_Te_5_, and the green lines are for (GST)_0.85_As_0.15_. The curves were obtained from the molecular dynamics production trajectories spanning over 40 ps.

**Figure 6:**
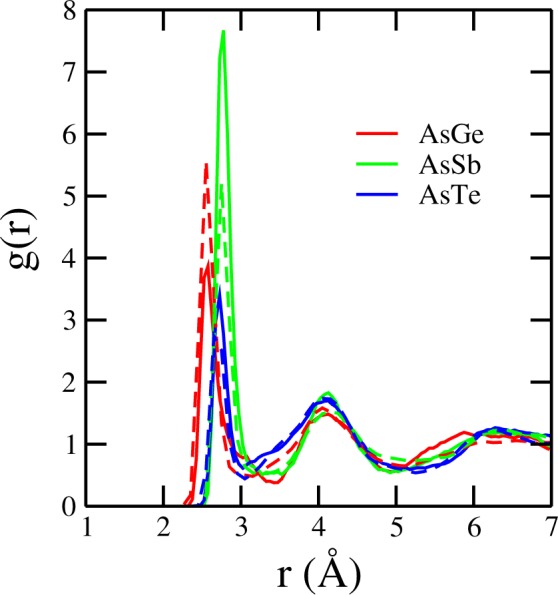
Pair correlation functions involving As and the other three elements of the Ge_2_Sb_2−0.3_As_0.3_Te_5_ (solid lines) and (GST)_0.85_As_0.15_ (dashed lines) model system.

In order to understand the difference between the amorphous structure of the As doped GST films we performed a first principles MD study of three extreme cases: one for the pure GST system, and two for alloys of GST having 3% and 15% of As following two approaches for the doping. To test the relative location of the different atomic species we first look at the pair distribution functions g(r) for all the pair of elements in the amorphous states. The results are displayed in Fig. 5 for the pairs not containing As, and in Fig. 6 for the pairs including As atoms.

First of all, we notice for the case of pure GST, the results reproduced fairly well the findings of Caravati and Bernasconi^34^. For the model Ge_2_Sb_2−0.3_As_0.3_Te_5_, which has 3% As doping by substitution of Sb atoms, the structure shows very small differences with respect to the model pure GST. The g(r) for Te-Te pairs, which is the majority component, is essentially unchanged by the addition of As. In the same way, for the Ge-Te and Sb-Te pairs, the g(r) undergo minimal changes. Consequently, we can safely conclude that the underlying Te based framework is robust and resist with minimal modifications the substitution of Sb atoms by As, at least up to a 3%. The pair distribution functions not involving Te do show some changes in the first and second peaks as the As atoms find their place in the amorphous structure. For the pure GST the total coordination around the Te atoms is 3.58, result of a split between 1.61 Ge, 1.36 Sb and 0.61 Te. For the Ge_2_Sb_2−0.3_As_0.3_Te_5_ the corresponding total coordination is 3.91, which is the result of 1.69 Ge, 1.32 Sb, 0.77 Te and 0.13 As. These coordination numbers are measured at the minimum of the g_Te-all_(r), which is r = 3.39 Å for the pure GST and r = 3.45 Å for the 3% As doped alloy. The substitution of some Sb by As atoms, which is smaller in size, results in a slight disruption around the Te structure that allows the atoms to accommodate having a larger coordination. A higher coordination is interpreted as a stronger glass.

The structural analysis of the model (GST)_0.85_As_0.15_ display a larger difference with respect to the pure systems than the 3% substituted doping model. The overall behavior of the g(r) show a structural rearrangement, in particular beyond the first peak. The total coordination around the Te atoms is 3.30 (calculated at the minimum r = 3.35 Å), results of 1.25 Ge, 1.06 Sb, 0.49 Te and 0.50 As.

In order to see the distribution of elements around the central As atoms for the two models for As doped GST, we display in Fig. 6 the corresponding pair distribution functions. The average coordination for As with Ge, Sb and Te is 0.78, 1.16 and 1.48 respectively for the Ge_2_Sb_2−0.3_As_0.3_Te_5_ model system. For the (GST)_0.85_As_0.15_ model the corresponding average coordination numbers are 0.77, 0.81 and 1.10. The change in coordination numbers with different As content reveals a change in the local structure.

**Figure 7:**
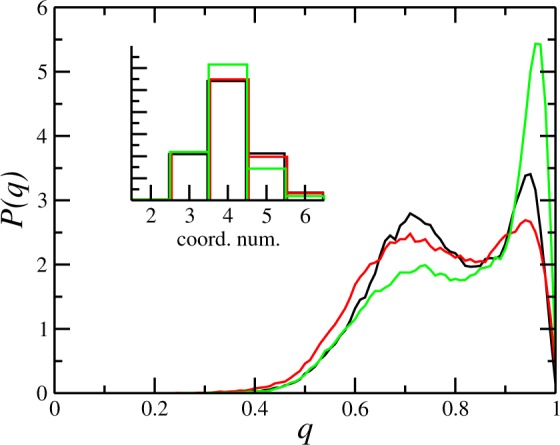
Probability distribution for the q order parameter, and total coordination numbers around the Ge atoms, calculated from the pure GST model (black lines), the As doped model with 3% Ge_2_Sb_2−0.3_As_0.3_Te_5_ (red lines), and 15% (GST)_0.85_As_0.15_ (green lines).

The coordination around the Ge atoms in the pure GST model is 5.0, and remains unchanged upon 3% substitution of Sb by As. For the (GST)_0.85_As_0.15_ model the coordination drops to 4.5, suggesting a tendency towards a stronger tetrahedral character in the amorphous state as As content increases. In order to check that, we analyze local q order parameter for the three simulated models. For a central atom *j*, *q* is defined as

$$q=1-\frac{3}{8}\sum _{i > k}{(\frac{1}{3}+\cos {\theta }_{ijk})}^{2}$$

where the sum runs over the atoms bonded to the central atom j and forming a bonding ${\theta }_{ij}$. In Fig. 7 we show q for central Ge atoms, with a cutoff at 3.2 Å, and only for those configurations with exactly four neighbors. We see that the pure GST system display a moderate structural order with the peak near the tetrahedral ideal q = 1. The substitution of Sb by As results in a disruption of the tetrahedral order. However, the proportional insertion of As while removing the other elements drives the system towards a high tetrahedral character, as shown by the strong peak near q = 1 (see Fig. 7).

**Figure 8:**
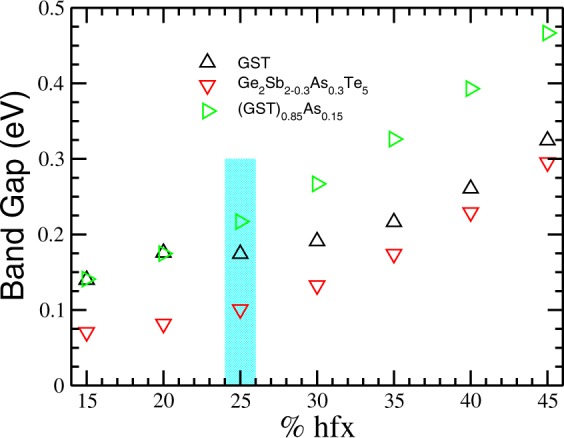
Band gaps for GST, Ge_2_Sb_2−0.3_As_0.3_Te_5_ and (GST)_0.85_As_0.15_ with varying % of Hartree-Fock exchange (%hfx).

In order to do a further test and elaborate a conclusion regarding the strength of the amorphous states in the three models, we calculate the total binding energy for each system. The final structures of the trajectories at 300 K were first quenched to their ground state. The procedure that we used follows the method of Guidon *et al*.^32,33^ that consist in varying the percentage of Hatree Fock Exchange (%hfx) from 15 to 45. In Fig. 8, we plot the results for the band gaps obtained for the three model systems. Our calculated band gap values for HSE06 (25%hfx) and HSE03 (30%hfx) are 0.18 and 0.19 eV for the GST system, respectively, in fair agreement with the value (0.2 eV) reported for a-GST by Akola *et al*.^41^ Fig. 8 shows that at 25%hfx and larger the band gap of the Ge_2_Sb_2−0.3_As_0.3_Te_5_ is smaller than the corresponding value for the pure GST. On the other hand, the band gap for (GST)_0.85_As_0.15_ is larger than that of pure GST, as in the experimental observations (see Table 1). This result suggest that proportional replacement of atoms is the better model to describe the experimentally obtained doped films.

The binding energy of the model systems is calculated as the total energy of relative to the total energy of their constituent individual isolated atoms. It was found that the difference in binding energy between Ge_2_Sb_2−0.3_As_0.3_Te_5_ and GST is −29 eV, and the corresponding difference between (GST)_0.85_As_0.15_ and GST is −51 eV. The fact the binding energy is stronger in As doped models supports the idea already obtained from the coordination analysis that the incorporation of As results in a glassy system with stronger bonds than in the pure GST case. As a consequence, the temperature needed to overcome the strength of the amorphous and crystallize the system is higher in the As doped alloys than in the pure GST case, in full agreement with the experimental findings.

### Summary

The dependence on As content on the optical and electrical properties of (GST)_1−x_As_x_ films were studied through the structural transition by annealing at different crystallization temperature. Although both, the pure GST and As doped GST are amorphous in the as-deposited state, only the GST alloy turns to crystalline NaCl-type structure after annealing at 150 °C for 1 h, and the doped films require annealing at higher temperatures. This indicates that As acts as a crystallization inhibitor and improves thermal stability. The optical band gap of the (GST)_1−x_As_x_ films decreases upon crystallization. GST alloys with 10% and 15% As addition show less optical contrast compared to un-doped GST due their higher amorphous character. The resistance of the as-deposited amorphous films and crystallized ones, both, increase with increasing As content. A larger resistivity of the crystalline phase will reduce the writing current and the higher crystallization temperature will significantly improves the retention time of the amorphous phase in a PCM device. The increased threshold voltage of 5.2 V for (GST)_0.85_As_0.15_ with respect to 4 V for GST verifies that a higher activation energy is required for the switching and indirectly reflects the strength of the amorphous network in the material. The additional bonds in the amorphous network, namely the Ge-As, Te-As and Sb-As support the finding for the greater activation energy found in the (GST)As films. This indicates that As creates higher thermal stability in the GST matrix and increases activation energy for transition in the system however with a cost of crystallization speed. First principles molecular dynamics were carried out on a GST model and two (GST) doped models at 3% and 15% of As (Ge_2_Sb_2−0.3_As_0.3_Te_5_ & (GST)_0.85_As_0.15_). The doped models have a stronger binding energy than the pure GST model, in line with the higher temperature required to achieve crystallization. The substitution of Sb by As decrease the tetrahedral character of the network. However, the proportional replacement of the GST atoms by As results in a clear enhancement of tetrahedral character.

## Supplementary information

Supplementary file
