# Supplementary material for: Crystallization properties of arsenic doped GST alloys
Source: Sci Rep. 2019 Sep 10;9:12985. doi: 10.1038/s41598-019-49168-z (PMC6737191; doi:10.1038/s41598-019-49168-z)
Supplement: Supplementary file 1 — Supplementary file [file 41598_2019_49168_MOESM1_ESM.docx]

**Supplementary Information**

**Crystallization properties of arsenic doped GST alloys**

Vinod E. Madhavan^1*^, Marcelo Carignano^1^, Ali Kachmar^1*^, K. S. Sangunni^2^

*^1^Qatar Environment and Energy Research Institute, Hamad Bin Khalifa University, Qatar Foundation, P. O. Box 34110, Doha, Qatar*

*^2^Department of Physics, Indian Institute of Science, Bangalore 560012, India*

*Corresponding authors ([vmadhavan@hbku.edu.qa](mailto:vmadhavan@hbku.edu.qa), [akachmar@hbku.edu.qa](mailto:akachmar@hbku.edu.qa))

**
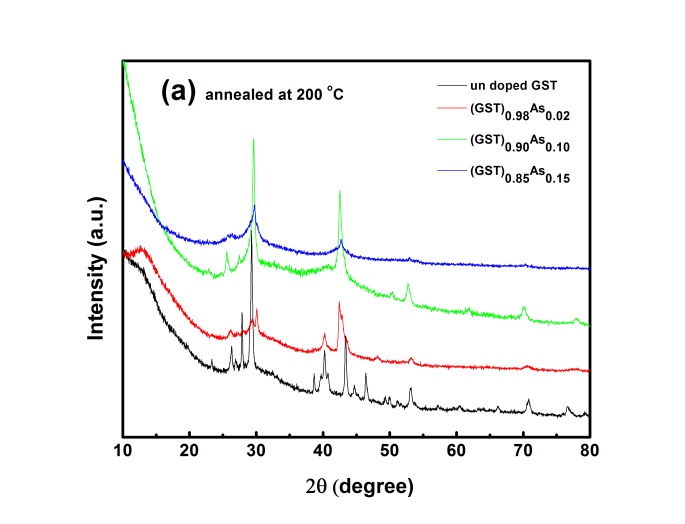
**

**
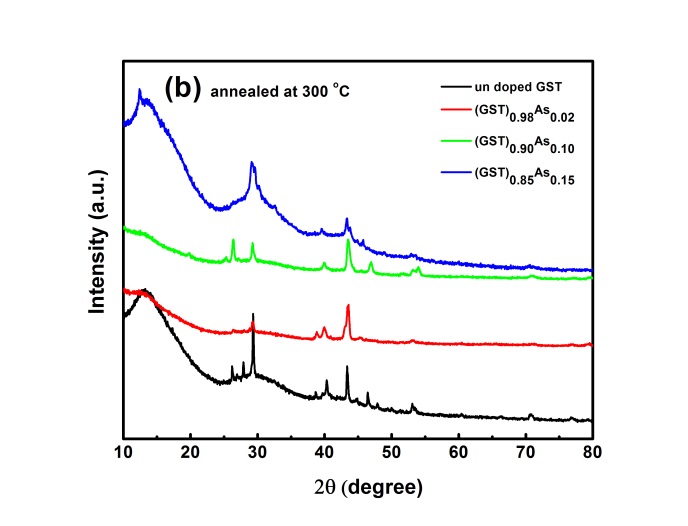
**

**Figure S1:** XRD of the (Ge_2_Sb_2_Te_5_)_1-x_As_x_ samples annealed (a) 200 ^o^C and (b) 300 ^o^C. The films annealed at 200 ^o^C are crystallized (Fi.g S1(a)). However, GST and As 0.02 at.% samples show a hexagonal structure formation whereas 0.10, 0.15 at.% arsenic doped sample show FCC structure. This is distinguishable by the absence of diffraction peak at 40.3 degree. This shows higher content of As suppresses the hexagonal formation. The arsenic suppresses the grain growth by sitting at the grain boundaries. At 300 ^o^C (Fig. S1(b)) all the samples are crystalline and hexagonal in structure.


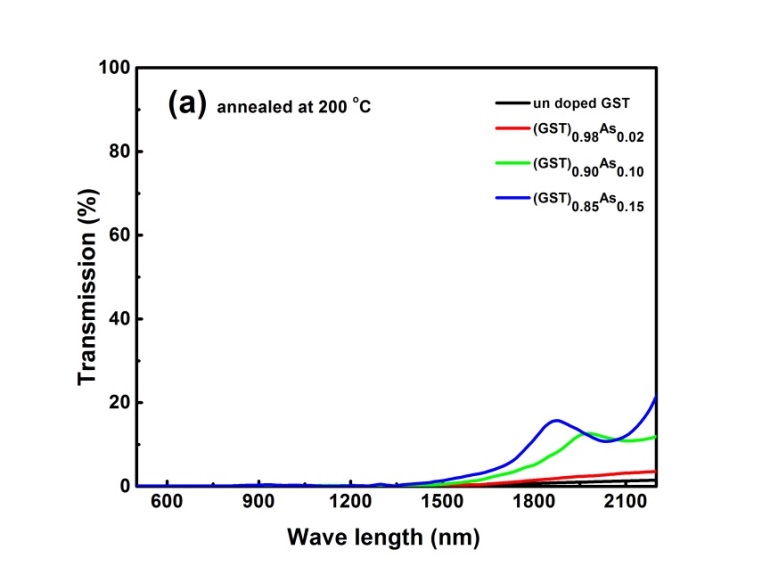


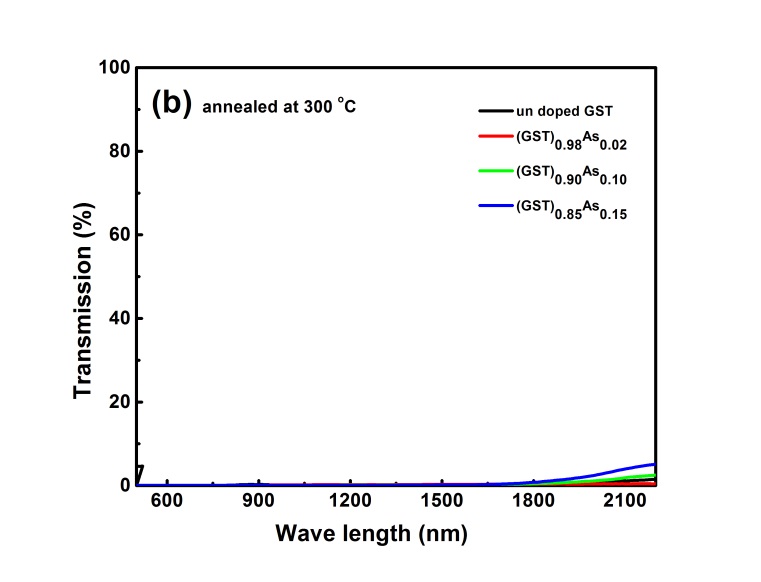


**Figure S2:** The VIS-NIR transmission spectra of the (Ge_2_Sb_2_Te_5_)_1-x_As_x_ samples annealed (a) 200 ^o^C and (b) 300 ^o^C. The maximum transmission is reduced as a result of crystallization in these samples.


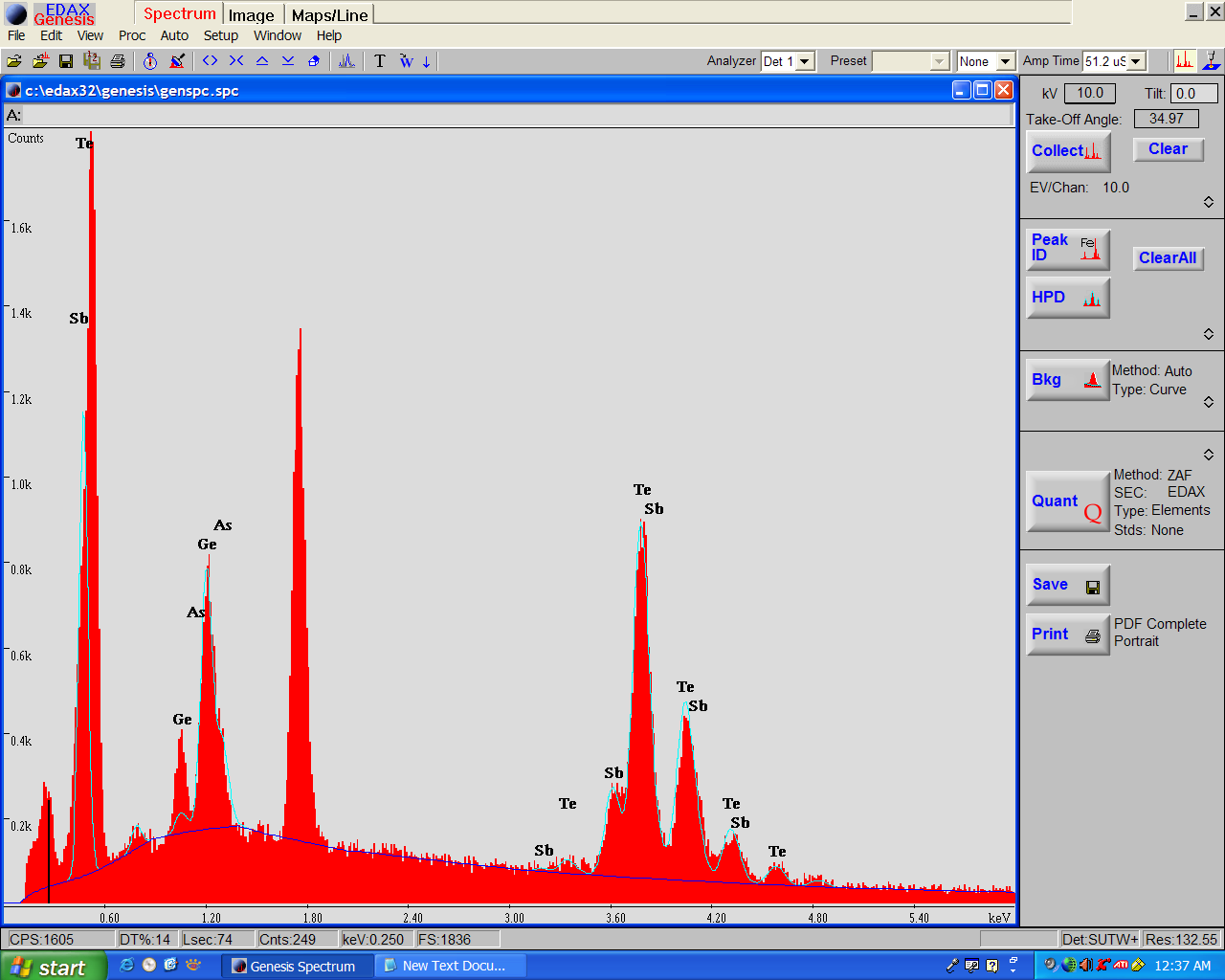


**Figure S3**: EDAX spectra of (GST)_90_As_10_ alloy.

| Elements | Ge | Sb | Te | As |
| --- | --- | --- | --- | --- |
| At% observed | 20.01 | 13.55 | 60.69 | 5.75 |
| At% Expected | 20 | 20 | 50 | 10 |

**Table S1**: Quantification table of (GST)_90_As_10_ table from EDAX analysis.
